# Notes and News

**Published:** 1905-04-29

**Authors:** 


### Notes and News.

Princess Christian laid the foundation-stone of the West Wales Sanatorium for Consumptives at Alltymynydd in Carmarthenshire.

The Governors of the Windsor and Eton Royal Dispensary and Infirmary have under discussion the site for the proposed new hospital.

The annual meeting of the Chelsea Hospital for Women will be held on Thursday, May 18th, at 4 p.m., the chairman, the Right Hon. the Lord Glenesk, presiding.

The fifteenth medical expedition is now organised by the Liverpool School of Tropical Medicine. The scene of action will be the Amazon River at its confluence with the Rio Negro.

Professor Osler has been succeeded at the Johns Hopkins University by Dr. Barker, lately professor of medicine at the Rush Medical College, Chicago. He is a graduate of Toronto.

Six cases of small-pox with two deaths are reported at Lewisham. Every precaution appears to be taken to avoid the further spread of the disease. The first case was unrecognised until other cases occurred, then the contacts were at once isolated.

A convalescent home for children has been opened at St. Anne's-on-Sea. It is the gift of Sir John Thursby and is intended for the use of patients from Burnley and East Lancashire. Sir John Thursby will maintain and endow the institution.

It is too early to form definite conclusions from the experience of sanatorium treatment, but the belief is very generally spreading that in the majority of cases temporary improvement and not cure must be looked for if the patient has to return to a life under the same conditions as before entering the sanatorium. Dr. Nevins, speaking at Liverpool recently, mentioned that statistics showed that 25 per cent. of sanatorium patients died within four years after their discharge.

We learn that cases of cerebro-meningitis are appearing in all parts of Germany.

The last patients have been discharged from the Dewsbury Small-pox Hospital, and the building is to be demolished.

A bazaar, in aid of the Metropolitan Hospital, will be held on May 9th and 10th at Surrey House, Marble Arch, by permission of Lord and Lady Battersea.

Lord Stanley will preside at the banquet in aid of the new out-patients' department of the St. John's Hospital for Diseases of the Skin on May 19th at the Savoy Hotel.

A sum of five hundred pounds has been presented by "a friend" to the Royal Victoria Hospital at Belfast, for the purpose of endowing a bed in remembrance of his mother.

The late Sir Edward Scott has bequeathed £5,000 each to the Walsall Hospital, the Birmingham General, and the Queen's Hospital, Birmingham, and to the County Hospital, Stafford.

A ball will be held on May 24th in aid of the Clarence Memorial wing at St. Mary's Hospital. Amongst the patronesses are the Princess of Wales, the Duchess of Fife, and Princess Henry of Battenberg.

The Southport Convalescent Hospital and Sea-Bathing Infirmary has received £5,000 through the will of the late Mr. James Holden, and this sum is to endow five beds in perpetuity for the use of Rochdale patients.

The Middlesbrough Isolation Hospital has been extended and two pavilions for 60 patients have been added; and at Hemlington a small-pox hospital has been established to contain 24 patients. This hospital is of corrugated iron.

A festival banquet in aid of the Great Northern Central Hospital will be held on May 17th, at the Savoy Hotel, under the presidency of the Duke of Connaught. The hospital needs a convalescent home and a children's ward, a new operating theatre, electrical equipment, and offices.

The new buildings at the Oldham Infirmary, which form a Jubilee Commemoration to Queen Victoria, consist of ward accommodation for 43 patients and extra staff, operating theatre, *x* ray and photographic rooms, and enlargement of the out-patient department. The cost amounted to upwards of £20,000.

The committee of the St. Paul's Eye and Ear Hospital, Liverpool, are in search of a site for a new hospital which will supersede the existing premises, but provide for the expanding needs of the institution. The hospital is now recognised by the Board of the Royal Colleges of Physicians and Surgeons, London, for the clinical teaching of ophthalmology, and is an integral part of the United Hospitals Medical School.

